# Aspirin for the prevention of cognitive decline in the elderly: rationale and design of a neuro-vascular imaging study (ENVIS-ion)

**DOI:** 10.1186/1471-2377-12-3

**Published:** 2012-02-08

**Authors:** Christopher M Reid, Elsdon Storey, Tien Y Wong, Robyn Woods, Andrew Tonkin, Jie Jin Wang, Anthony Kam, Andrew Janke, Rowan Essex, Walter P Abhayaratna, Marc M Budge

**Affiliations:** 1 School of Public Health & Preventive Medicine, Monash University, Melbourne Australia; 2 Department of Neuroscience (Medicine), Monash University, Melbourne, Australia; 3 Centre for Eye Research Australia, Royal Victorian Eye and Ear Hospital, University of Melbourne, Australia; 4 Singapore Eye Research Institute, National University of Singapore, Singapore; 5 College of Medicine, Biology and Environment, Australian National University, Canberra, Australia; 6 Centre for Vision Research, University of Sydney, Australia

## Abstract

**Background:**

This paper describes the rationale and design of the ENVIS-ion Study, which aims to determine whether low-dose aspirin reduces the development of white matter hyper-intense (WMH) lesions and silent brain infarction (SBI). Additional aims include determining whether a) changes in retinal vascular imaging (RVI) parameters parallel changes in brain magnetic resonance imaging (MRI); b) changes in RVI parameters are observed with aspirin therapy; c) baseline cognitive function correlates with MRI and RVI parameters; d) changes in cognitive function correlate with changes in brain MRI and RVI and e) whether factors such as age, gender or blood pressure influence the above associations.

**Methods/Design:**

Double-blind, placebo-controlled trial of three years duration set in two Australian academic medical centre outpatient clinics. This study will enrol 600 adults aged 70 years and over with normal cognitive function and without overt cardiovascular disease. Subjects will undergo cognitive testing, brain MRI and RVI at baseline and after 3 years of study treatment. All subjects will be recruited from a 19,000-patient clinical outcome trial conducted in Australia and the United States that will evaluate the effects of aspirin in maintaining disability-free longevity over 5 years. The intervention will be aspirin 100 mg daily versus matching placebo, randomized on a 1:1 basis.

**Discussion:**

This study will improve understanding of the mechanisms at the level of brain and vascular structure that underlie the effects of aspirin on cognitive function. Given the limited access and high cost of MRI, RVI may prove useful as a tool for the identification of individuals at high risk for the development of cerebrovascular disease and cognitive decline.

**Trial Registration:**

clinicaltrials.gov Identifier: NCT01038583

## Background

Cognitive decline and dementia are major causes of morbidity and mortality worldwide and are substantially burdensome to the affected persons, their caregivers, and society in general [1]. Despite extensive research over the past 20 years, there remain important and formidable challenges to conducting research, particularly into the area of prevention. A recent National Institutes of Health Consensus Development Conference concluded that large-scale population-based studies and randomised controlled trials are critically needed to investigate strategies to maintain cognitive function in individuals at risk or those experiencing cognitive decline [1]. The Aspirin in Reducing Events in the Elderly (ASPREE) Study is one such large scale trial and this manuscript describes a neurovascular imaging sub-study (ENVIS-ion) that will explore the possible mechanisms through which aspirin may act to influence cognitive function.

### Rationale for the ENVIS-ion Study

A decline in cognitive abilities is commonly held to be an inevitable consequence of aging with greater degrees of decline suggesting the superimposition of progressive neuropathology. Even in healthy older adults, a small but definite age-related decrease in performance on executive function, verbal fluency, verbal memory, and cognitive speed tasks is evident [2], but the mechanisms leading to decline are not completely understood [3]. Similarly, reports of the effects of preventive interventions on the trajectories of changes of brain structure and cognitive performance with aging and early dementia are scarce. Nonetheless, it is clear that in adults over 70 years of age with declining cognitive performance, up to 15% per year will cross the threshold for the diagnosis of clinical dementia [4,5].

Ischaemia of brain white matter may cause cognitive decline in its own right, but it is also present in the majority of those with Alzheimer's (AD). Vascular risk factors have emerged as important contributors to the development of AD and to sub-clinical brain infarcts/ischaemia. This is the most common pathology found in community-based studies of older adults with cognitive decline [6,7]. Therefore, vascular disease is an attractive target for primary preventive therapy.

The question of whether low-dose aspirin might be protective against cognitive decline, presumably via an effect on platelet aggregation, remains unanswered. Several community-based, prospective studies and a cross-sectional study have suggested an association of aspirin treatment with preservation of either episodic memory or global cognitive performance [8]. Further studies have contradicted these results and found that long-term use of aspirin does not provide benefits for cognition among generally healthy women aged 65 years or over and may even increase the risk of development of AD [9,10].

However a number of studies have demonstrated the effective use of aspirin in the prevention of other conditions such as stroke, coronary heart disease events and diabetes. Aspirin has been shown to reduce the risk of serious vascular events (non-fatal myocardial infarction, non-fatal stroke, or vascular death) by about one quarter, in high-risk patients [11].

### Magnetic Resonance Imaging (MRI)

Brain MRI changes correlate with white matter ischaemia/infarction and MRI is commonly used to investigate the underlying mechanisms and predictors of dementia and cognitive decline [7]. MRI can assess brain structure changes using volumetric tissue measurements, or intensity-based lesion identification to quantify structural changes such as hyper-intense white matter lesions (WMH) or infarcts. The association of greater WMH volume with poorer cognitive performance remains a focus of ongoing research. While some studies have found that WMH presence and volume rather than location determines the presence of executive dysfunction in those who are non-demented, others have specifically related periventricular WMH to such cognitive dysfunction [12]. Silent brain infarcts (SBI) are frequently seen on MRI in healthy older adults and are associated with dementia incidence and cognitive decline as well as subsequent clinical stroke [13,14]. The Rotterdam Stroke Study found that the presence of SBIs at baseline more than doubled the risk of dementia [14]. In addition, the presence of SBIs at baseline was associated with poorer initial cognitive performance and a steeper decline in global cognition. Also, the incidence of SBI increased markedly with age and a prevalent SBI at baseline strongly predicted a new silent infarct on the second MRI [15]. The authors concluded that the cardiovascular risk factors for SBI are similar to those for stroke.

The preventive effects of aspirin on MRI-detected SBI and WMH have previously been investigated. A study by Sato et al. found that in patients with non-valvular atrial fibrillation (NVAF), and perhaps also those in sinus rhythm, aspirin may reduce SBIs [13]. Fujita et al. also reported a significant reduction in progression of WMH over 4 years with aspirin treatment for those with corrected platelet hyper-aggregability in a study of older, treated patients compared with age- and WMH-matched controls [10]

### Retinal Vascular Imaging

While brain MRI can potentially provide an efficient surrogate for cognitive decline and dementia, it is costly and not widely accessible, making it impracticable for population screening. Retinal vessels share many features with the cerebral vasculature. Hence, should changes in the two be highly correlated, retinal digital photography through non-dilated pupils would provide the opportunity to combine a relatively inexpensive, rapid, widely available tool with automated analysis to assess cerebrovascular pathology and inform selection of those likely to benefit most from further investigation and preventive therapies.

It is well known that clinically detectable changes in retinal blood vessels (retinal arteriolar narrowing, arteriovenous nicking, microaneurysms, retinal hemorrhages and cotton wool exudates) are markers of the effects of chronic hypertension and other vascular processes [16,17]. Because the retinal circulation can be viewed non-invasively, it offers a unique insight into the cerebral microcirculation in vivo and may allow a greater understanding of cerebrovascular pathophysiology [16]. Retinal and cerebral arteriolar histopathologies are highly correlated in those who have died of stroke, while retinal microvascular flow is reduced in persons with WMH and SBI on MRI [18].

Large population-based studies have shown that retinal vascular changes are associated with subclinical and clinical cerebrovascular disease, including cognitive impairment [16,19]. Other studies have demonstrated that changes in RVI parameters have been associated with poorer cognitive performance in people with diabetes [15,20]. In the Atherosclerosis Risk in Communities (ARIC) study, RVI changes on photomicrography were associated not only with incident stroke, but also with subclinical changes in MRI-defined parameters, including cerebral infarction, WMH (both sulcal and periventricular) and atrophy [21-23]. Furthermore, the ARIC study demonstrated that in patients without a previous stroke, retinal vascular changes were associated with poorer cognitive function independent of traditional risk factors [21]. However this study was unable to correlate changes in cognition with RVI changes over time. Similar studies have also examined the relationship between RVI changes and WMH volume [24]. Retinal arterial pathology, especially retinal arterial sclerosis and narrowing, has also been shown to correlate with MRI signs of cerebral small-vessel disease in both hypertensive and normotensive subjects [25]. In summary, there are few studies that have examined the effect of aspirin on MRI-detected SBI and WMH and RVI parameters and their relationship to cognition. Importantly, RVI may be a relatively inexpensive, more widely available imaging tool that could be used to screen and predict the onset and progression of subclinical cerebrovascular disease.

### The ENVIS-ion trial

The ENVIS-ion trial is designed to determine whether;

a) low-dose aspirin reduces the development of WMH and SBI as assessed by MRI,

b) a reduction in changes in RVI parameters is observed with low-dose aspirin therapy,

c) changes in RVI parameters parallel changes in brain MRI,

d) baseline cognitive function correlates with baseline MRI and RVI parameters,

e) changes in cognitive function correlate with changes in brain MRI and RVI, and

f) factors such as age, gender, education or blood pressure influence the above associations.

## Methods/design

ENVIS-ion is a multi-center, three-year randomized, double-blind, placebo-controlled trial in 600 healthy adults without dementia recruited from general practices in Melbourne and Canberra, Australia. ENVIS-ion is a sub-study of the Aspirin in Reducing Events in the Elderly (ASPREE) clinical trial.

### The ASPREE Trial

ASPREE is a large scale, randomized double-blind placebo-controlled trial of 100 mg enteric-coated aspirin daily, which is examining the benefits and risks of such treatment in apparently healthy subjects from Australia and the United States aged 70 years or more without overt cardiovascular disease or dementia. Although low-dose aspirin has been shown to reduce the risk of vascular events in a wide range of primary and secondary care settings [6,26,27], the benefit of aspirin may be offset by a variety of adverse effects, such as gastrointestinal and intracranial hemorrhage; the balance of risks and benefits has not been established in older subjects.

### Subjects

Participants for the ASPREE trial and ENVIS-ion sub-study will be recruited from general medical practices and community advertising. General practice participants will be sent a letter inviting them to participate in the study. Following preliminary telephone screening, participants will be included in the study if they are aged 70 years of age or over, not institutionalized, provide informed consent, and are physically capable of attending their family physician and for ENVIS-ion, the MRI facility. Participants in the ASPREE parent study will be excluded if they have a history of a previous cardiovascular event, a serious inter-current illness likely to cause death within the next 5 years, a current or recurrent condition with a high risk of major bleeding, anaemia, an absolute contraindication to or allergy to aspirin, current continuous use of aspirin or other anti-platelet drug or anticoagulant, a systolic blood pressure ≥180 mmHg or diastolic blood pressure ≥105 mmHg, a history of dementia or a Modified Mini-Mental State Examination (3 MS) score ≤77, an inability to perform any one of the six Katz ADL's [28] independently or with more than 'a little difficulty', pill-taking compliance outside the range of 80-120% during placebo run-in phase, or are currently participating in another clinical trial. All ASPREE participants who have no known contraindications to MRI and can attend the MRI facility are deemed eligible for entry into the ENVIS-ion sub-study. MRI contraindications include a) cardiac pacemaker, b) non-compatible cerebral-aneurysm clip, c) cochlear implant, d) retained metal fragment in the eye and e) stapedectomy with implant. Treatment allocation will remain blinded to investigators and subjects until the conclusion of the main ASPREE Study.

### Consent and Ethics Approval

All participants will consent separately for the ENVIS-ion trial after ASPREE trial consent. Protocols (etc.) have been approved for both studies by the Monash University Health and Research Ethics Committee.

### Assessments

All participants will complete a baseline and 3-year follow-up assessment which will include ASPREE plus additional ENVIS-ion cognitive testing, MRI and RVI. Additionally, participants will complete annual ASPREE follow-up visits comprised of compliance checking by pill count, blood pressure measurement, reassessment of behavioural traits, cognitive and quality-of-life tests and biochemical markers as part of the ASPREE study (Table 1). Retention will be enhanced through telephone contact quarterly along with regular newsletter correspondence. The three year follow-up time was chosen as an optimal time-frame to allow for the potential impact of treatment and minimize loss to follow-up due to major events such as death.

**Table 1:** Measurement schedule for the participants of the ENVIS-ion trial.

	Recruitment, Screening & Baseline	Annualreview (1 yr)	Annualreview (3 yr)	Annualreview (5 yr)
**ENVIS-ion study**				
Brain MRI & RVI	X		X	
Additional cognitive function tests^a^	X		X	
**ASPREE parent study**				
Inclusion/exclusion criteria; informed consent	X			
Cognitive function tests^b^	X	X	X	X
Demographics & physical measures^c^	X	X	X	X
Blood pressure & cardiovascular biomarkers^d^	X	X	X	X
Health behaviors & lifestyle measurements^e^	X	X	X	X

^a ^Stoop test (Victoria version) & Color Trails test ^b ^3 MS, Digit Symbol Modalities test, COWAT, and Hopkins Verbal hearing test ^c ^Includes first language, level of education, height, weight, abdominal circumference, family history & co-morbidity; ^d ^Includes total cholesterol, LDL-Cholesterol, HDL-Cholesterol, triglycerides, C-Reactive Protein, Hemoglobin, fasting glucose & creatinine;^e ^Includes physical activity, smoking history & alcohol use, SF-36 & IADL

### Data acquisition and storage

Data will be stored securely, anonymized by trial number, with the trial number - identity key stored separately.

### Study Medications and supplies

An initial box with placebo medication for 4 weeks will be given to participants at the screening visit to check medication compliance. If for whatever reason a participant cannot attend their randomization visit at 4 weeks, they will receive another screening visit and supply of placebo. Participants in the study will be allocated to one of two treatments: a) acetylsalicylic acid (ASA) 100 mg, administered as an enteric-coated white tablet or b) placebo, an enteric-coated white tablet with identical appearance. A 100 mg dosage was selected as this is the common international dose. Enteric coating will ensure that both the active and placebo medication have an identical taste. Randomization of drug and placebo will be achieved via interactive voice recognition software (IVRS) and will be stratified by primary care practice and age. Subsequent to the randomization visit each participant will be provided with 12 months' supply of study drug; either aspirin or placebo. At each annual visit each participant will be provided with the next 12 months supply of their allocated drug. ENVIS-ion participants will continue receiving randomized therapy for the remaining two years of the ASPREE main trial after completing their involvement in the ENVIS-ion study.

### Cognitive testing

Cognitive testing for the parent ASPREE trial consists of i) the Modified Mini-mental State examination as a test of general intellectual function, on which participants must score > 77/100 at entry [29]; ii) a single-letter ("F") Controlled Oral Word Association test (COWAT), which gives equivalent information to the longer 3-letter tests, as a measure of fluency [30]; iii) the Hopkins Verbal Learning Test-Revised as a measure of verbal anterograde episodic memory [31]; and iv) an alternative version of the Digit-Symbol Modalities test as a measure of psychomotor speed [32]. The Center for Epidemiological Studies - Depression Scale (CES-D) is also included in the ASPREE battery for later analysis as a covariate [33]. In addition to the cognitive function testing in ASPREE, ENVIS-ion participants will complete the Stroop test (Victoria version) as a measure of cognitive inhibition, and the Color Trails test, which is a non-language based test of sequencing, alternation and processing speed [32,34-36].

The emphasis on aspects of executive functioning (COWAT, Stroop, Color Trails) and processing speed (SDMT, Color Trails) reflects the typical neuropsychological pattern seen with cognitive impairment due to subcortical ischaemic damage [37], the focus of this study. The choice of test battery is based on the neuropsychological batteries suggested for assessment of vascular cognitive impairment [38]. Assessors will undergo standard training and assessment of the administration of tasks and this will be recertified on an annual basis and if the number of assessments falls below three in a three month period.

### Magnetic Resonance Imaging

A non-contrast MRI examination will be performed on all participants at either the Canberra or Alfred Hospitals at baseline and 3 years later using 1.5 Tesla Siemens and GE machines respectively. A number of standardized MRI Brain sequences will be acquired to allow morphologic, microstructure and functional assessment. First, a volumetric 3D T1-weighted sequence is used to obtain high resolution anatomical information. This is followed by the FLAIR and T2 sequences used to assess inflammation and signal alteration in brain tissues. A Gradient Echo T2-weighted (GE T2) or Susceptibility Weighted Imaging (SWI) sequence will be used to detect micro-hemorrhages. Where technically possible, Arterial Spin Labeling (ASL) will be used to quantify brain perfusion without the need to use exogenous contrast agents. Finally, Diffusion Tensor Imaging (DTI) data will be acquired to evaluate underlying neural tissue diffusion properties, permitting assessment of complex brain microstructure (e.g. axonal loss in white matter tracts).

MRI analyses will be performed at the Dementia Collaborative Research Centre Imaging Laboratory in Canberra. All assessment will be conducted blinded to treatment allocation and participant data. Standard pre-processing for all volumetric images will follow best practice that has been developed for such studies with large numbers of participants [39]. This includes; 1) N3 B0 MRI nonlinear intensity distortion correction, between-scan intensity normalization and registration to a population-specific model that is then later matched to the ICBM model of cortical anatomy; 2) model-based segmentations of whole brain, ventricular, lobar and hippocampal volumes; 3) white matter/grey matter (WM/GM) segmentation: a multi-spectral segmentation utilizing all acquired data types will provide a good measure of the CSF and WM/GM volumes, and consequently, accurate atrophy measures as well as lobar WM/GM differences; 4) nonlinear registration of the EPI T2 from the functional scans to the gradient echo volumetric T2. The GE T2 will then be matched to the volumetric T1 and then to the ICBM model using an iterative nonlinear registration to reduce within-group subject differences at time steps.

Calculations will be made from the scans regarding WMH with respect to their total volume and rate of change in volume. The presence or absence of sub-cortical infarction will be determined and the total brain volume calculated. The presence and count of cerebral micro-bleeds will be determined from the GE T2 and SWI data and brain tissue perfusion will be assessed from the ASL scans. Volumetric change of structures including the hippocampus and ventricles will be assessed via the automated comparison of each of the individuals to average models of anatomy.

### Retinal Vascular Imaging

**Figure 1:**
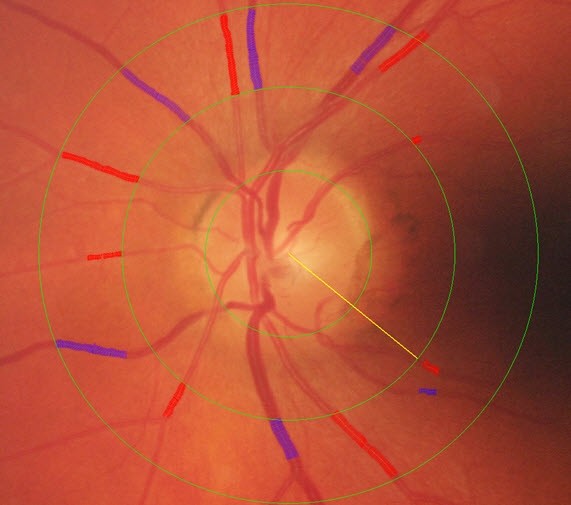
**Retinal Vascular Imaging measurement of Retinal Calibre**.

Two color retinal photographs, centered on the optic disc (ETDRS standard fields 1 (Figure 1)) and macula (standard field 2), respectively, will be taken from each eye of the participant. The photographs will be taken after 5 minutes of dark adaption, without pharmacological pupil dilation, using a Canon NMR 45 digital fundus camera. Photographers are certified for quality of measurement by the central reporting group prior to the commencement of the study.

RVI images will be sent to the core analysis laboratory at the Retinal Vascular Imaging Centre in Melbourne for analysis by trained graders. Photographs centered on the optic disc of each eye will be examined using standard computer-assisted retinal analysis software that was initially developed for the ARIC study [40]. The grading approach measures retinal vessel diameters and combines the measurements into central retinal arteriolar and venular equivalents (CRAE and CRVE) with formula adjusting for branching, following the Parr and Hubbard formula [40]. The analysis software has been modified and improved, and used in a number of studies including the Australian Diabetes, Obesity and Lifestyle Study (AusDiab) and the Blue Mountains Eye Study (BMES) in Australia [41]. The graders will use an automated system to measure the calibers of all arteriolar and venular branches crossing a zone defined as the region between 1/2 and 1 disc diameter from the optic disc. The grader will monitor the whole process, carefully select appropriate vessel regions for caliber measurement and correctly identify small arteriolar and venular branches. Correction factors are used in analysis of the measured values of absolute vessel caliber. All grading will be conducted blinded to treatment allocation. Technically unsatisfactory scans are typically not improved by re-photography, and will not be included in the analysis.

The qualitative presence of focal retinal arteriolar narrowing, arteriovenous nicking, arteriolar wall opacity ("silver wiring") and the presence of retinopathy lesions will also be assessed. Each lesion is classified as definite, questionable/probable, or none in each of the retinal photographic fields.

### Sample size

Approximately 300 participants each from Melbourne and from Canberra, Australia will be enrolled in ENVIS-ion. The sample size calculations were based on a) a pilot study of 144 older adults, in which acceptance of MR imaging was 88%, and which produced MR imaging estimates of change in relevant structural brain parameters over a two-year period; b) rate of change of WMH over 3 years from the PROSPER trial of adults aged 70 years or over [42]; and c) cautious interpretation of the report by Sato et al., in which treatment with around 300 mg/day of aspirin over one year reduced incident SBIs on MRI from 20.6% to 9.6% in 150 participants with non-valvular atrial fibrillation (i.e. a relatively higher risk group than in ENVIS-ion) [13,43]. Based on power calculations related to the primary hypothesis, we conservatively estimate the need for at least 230 per treatment arm to achieve a statistical power of 0.9 to show a relative risk reduction of 15% with a 2-sided α of 0.05.

### Statistical analysis

Group descriptive statistics will be used to define the baseline characteristics of subjects randomized to aspirin and placebo groups. Continuous variable changes will be normalized prior to analysis and non-parametric methods will be used where required. Changes in continuous variables for both MRI and RVI over three years will be analyzed using analysis of covariance (ANCOVA) adjusting for baseline values (Aims 1,2 & 5). Chi-square and logistic regression analysis will be used to assess changes in categorical variables. Correlation and regression analysis will be used to determine the association between changes in MRI and RVI measures and the association of baseline cognitive measures with MRI and RVI parameters (Aims 3 & 4). Generalized linear models will be developed to determine whether age, gender, education, previous aspirin use and hypertensive status modify the treatment effects on MRI and RVI outcome measures (Aim 6).

## Discussion

While the ASPREE trial will address the question of whether low-dose aspirin can prevent death or the onset of disability, including dementia and cognitive decline, over a 5-year period in people 70 years and over, the ENVIS-ion sub-study provides a unique opportunity to examine whether regular low-dose aspirin reduces the rate of increase of brain MRI-measured WMH and SBI volumes. In addition, the trial will also investigate if changes in RVI parameters parallel brain MRI changes over 3 years and explore the value of MRI and RVI in assessing and defining vascular changes and cognitive function. The ASPREE trial and the ENVIS-ion sub-study target healthy adults over the age of 70 years living in the community: this large and growing segment of the population needs to be the focus of specific attention as cognitive function will be a major determinant of their overall health status and the extent of assistance that will be required during their projected average life expectancy of 10 to 15 years [6].

Undeniably, although we currently deploy more resources for those with greater morbidity, it is potentially *healthy *older adults who have most to gain from preventive strategies. The thorough evaluation of an inexpensive, widely available intervention such as low-dose aspirin for the prevention of dementia and cognitive decline is timely in the context of the ever-increasing numbers of older people and the associated rising incidence of dementia.

The unique features of the ENVIS-ion study lie in the combination of the extensive but targeted nature of the cognitive assessment, combined with the detailed assessment of the brain and retina. Evidence shows that retinal micro-vascular changes reflect cerebral micro-vascular changes in aging, as well as in diseases such as vascular dementia and stroke [44]. Given the limited access and high cost of MRI, and the increasing penetration of RVI into routine ophthalmic and optometric practice, RVI may prove useful as a tool for the identification of individuals at high risk for the development of cerebrovascular disease and cognitive decline, who can then be targeted for intensive risk factor reduction, possibly including aspirin therapy.

The ENVIS-ion study will add to the current knowledge in the field by identifying whether aspirin treatment can influence the development and progression of white matter hyper-intensities which in-turn influence cognitive function. Importantly, it will determine whether retinal vascular imaging, a much less expensive approach, may be an alternative approach to MRI measurement. Clinically this may result in a low cost alternative to identify whether older people who may benefit from aspirin therapy, should be treated. Whilst there are a number of ongoing trials exploring the changes in white matter lesions and associations with cognitive function in ageing, none are looking at the association between retinal vascular imaging and MRI changes over time and their relationship with cognitive function [45].

A potential limitation of the study is that it will only provide information on people who reach the age of 70 years free from manifest cardiovascular disease and cancer, although most individuals will probably have subclinical atherosclerosis. However, given the increases in lifespan projected over the future decades, this is likely to represent an ever-increasing number of older people. The three year follow-up may be considered as a limitation; however it may also be optimal for the effects of aspirin to be demonstrable on the vasculature while minimizing potential loss to follow-up.

Lifestyle or pharmacological interventions that minimize decline in cognitive performance are urgently required. Aspirin is an appealing therapeutic candidate given its universal availability, community acceptance and low cost, all of which ensure equity and would facilitate community uptake. Equally, analyses of the brain and automated retinal imaging results from this trial will inform guidelines for health professionals where currently uncertainty exists. The ENVIS-ion trial will significantly inform global efforts to maximize our abilities to "maintain the brain" and, in addition, may improve the understanding of the mechanisms at the level of brain and vascular structure that underlie the effects of aspirin on cognitive function.

## Competing interests

The authors declare that they have no competing interests.

## Authors' contributions

CMR, ES, TYW, RW, AT, WPA and MMB designed and planned all aspects of the proposed ENVISION Study. JJW, AK, AJ and RE are involved in image data capture and analysis. All authors read and approved the final manuscript. The project has been supported through the NHMRC Project Grant number 417460 awarded to MMB, ES, AT, TYW,CMR and WPA.

## Pre-publication history

The pre-publication history for this paper can be accessed here:

http://www.biomedcentral.com/1471-2377/12/3/prepub

## References

[B1] Daviglus ML Bell CC Berrettini W Bowen PE Connolly ES Cox NJ Dunbar-Jacob JM Granieri EC Hunt G McGarry K National Institutes of Health State-of-the-Science Conference Statement: Preventing Alzheimer's Disease and Cognitive Decline NIH State-of-the-Science Conference Statement 2010 27 4 1 30 20445638

[B2] Van Hooren SAH Valentijn AM Bosma H Ponds RWHM Van Boxtel MPJ Jolles J Cognitive functioning in healthy older adults aged 64-81: A cohort study into the effects of age, sex, and education Aging, Neuropsychology, and Cognition 2007 14 1 40 54 10.1080/138255890969483 17164189

[B3] Driscoll I Resnick SM Troncoso JC An Y O'Brien R Zonderman AB Impact of Alzheimer's pathology on cognitive trajectories in nondemented elderly Annals of Neurology 2006 60 6 688 695 10.1002/ana.21031 17192929

[B4] Hsiung GYR Donald A Grand J Black SE Bouchard RW Gauthier SG Loy-English I Hogan DB Kertesz A Rockwood K Outcomes of cognitively impaired not demented at 2 years in the Canadian Cohort Study of Cognitive Impairment and Related Dementias Dementia and Geriatric Cognitive Disorders 2006 22 5-6 413 420 10.1159/000095751 16966831

[B5] Piguet O Grayson DA Creasey H Bennett HP Brooks WS Waite LM Broe GA Vascular risk factors, cognition and dementia incidence over 6 years in the Sydney Older Persons Study Neuroepidemiology 2003 22 3 165 171 10.1159/000069886 12711848

[B6] Ince PG Pathological correlates of late-onset dementia in a multicentre, community-based population in England and Wales Lancet 2001 357 9251 169 175 11213093 10.1016/s0140-6736(00)03589-3

[B7] Clarke R Joachim C Esiri M Morris J Bungay H Molyneux A Budge M Frost C King E Barnetson L Leukoaraiosis at presentation and disease progression during follow-up in histologically confirmed cases of dementia 2000 903 497 500 10.1111/j.1749-6632.2000.tb06405.x 10818544

[B8] Shepherd CE Piguet O Broe GA Creasey H Waite LM Brooks WS Kril JJ Histocompatibility antigens, aspirin use and cognitive performance in non-demented elderly subjects Journal of Neuroimmunology 2004 148 1-2 178 182 10.1016/j.jneuroim.2003.11.007 14975599

[B9] Kang JH Cook N Manson J Buring JE Grodstein F Low dose aspirin and cognitive function in the women's health study cognitive cohort British Medical Journal 2007 334 7601 987 990 10.1136/bmj.39166.597836.BE 17468120 PMC1867896

[B10] Fujita S Kawaguchi T Uehara T Fukushima K Progress of leukoaraiosis is inhibited by correction of platelet hyper-aggregability International Psychogeriatrics 2005 17 4 689 698 10.1017/S104161020500164X 16271159

[B11] Antithrombotic Trialists C Aspirin in the primary and secondary prevention of vascular disease: collaborative meta-analysis of individual participant data from randomised trials The Lancet 2009 373 9678 1849 1860 10.1016/S0140-6736(09)60503-1 PMC2715005 19482214

[B12] Bracco L Piccini C Moretti M Mascalchi M Sforza A Nacmias B Cellini E Bagnoli S Sorbi S Alzheimer's disease: Role of size and location of white matter changes in determining cognitive deficits Dementia and Geriatric Cognitive Disorders 2005 20 6 358 366 10.1159/000088562 16192726

[B13] Sato H Koretsune Y Fukunami M Kodama K Yamada Y Fujii K Kitagawa K Hori M Aspirin attenuates the incidence of silent brain lesions in patients with nonvalvular atrial fibrillation Circulation Journal 2004 68 5 410 416 10.1253/circj.68.410 15118280

[B14] Vermeer SE Den Heijer T Koudstaal PJ Oudkerk M Hofman A Breteler MMB Incidence and risk factors of silent brain infarcts in the population-based Rotterdam Scan Study Stroke 2003 34 2 392 396 10.1161/01.STR.0000052631.98405.15 12574548

[B15] Ryan CM Geckle MO Orchard TJ Cognitive efficiency declines over time in adults with Type 1 diabetes: Effects of micro- and macrovascular complications Diabetologia 2003 46 7 940 948 10.1007/s00125-003-1128-2 12819900

[B16] Wong TY Is retinal photography useful in the measurement of stroke risk? Lancet Neurology 2004 3 3 179 183 10.1016/S1474-4422(04)00682-9 15029894

[B17] Wong T Mitchell P The eye in hypertension Lancet 2007 369 9559 425 435 10.1016/S0140-6736(07)60198-6 17276782

[B18] Schneider R Rademacher M Wolf S Lacunar infarcts and white matter attenuation: Ophthalmologic and microcirculatory aspects of the pathophysiology Stroke 1993 24 12 1874 1879 10.1161/01.STR.24.12.1874 8248970

[B19] Cooper LS Wong TY Klein R Sharrett AR Bryan RN Hubbard LD Couper DJ Heiss G Sorlie PD Retinal microvascular abnormalities and MRI-defined subclinical cerebral infarction: The atherosclerosis risk in communities study Stroke 2006 37 1 82 86 16306463 10.1161/01.STR.0000195134.04355.e5

[B20] Ding J Patton N Deary IJ Strachan MWJ Fowkes FGR Mitchell RJ Price JF Retinal microvascular abnormalities and cognitive dysfunction: A systematic review British Journal of Ophthalmology 2008 92 8 1017 1025 10.1136/bjo.2008.141994 18614569

[B21] Wong TY Klein R Sharrett AR Nieto FJ Boland LL Couper DJ Mosley TH Klein BEK Hubbard LD Szklo M Retinal microvascular abnormalities and cognitive impairment in middle-aged persons: The Atherosclerosis Risk in Communities Study Stroke 2002 33 6 1487 1492 10.1161/01.STR.0000016789.56668.43 12052979

[B22] Wong TY Klein R Couper DJ Cooper LS Shahar E Hubbard LD Wofford MR Sharrett AR Retinal microvascular abnormalities and incident stroke: The Atherosclerosis Risk in Communities Study Lancet 2001 358 9288 1134 1140 10.1016/S0140-6736(01)06253-5 11597667

[B23] Wong TY Klein R Sharrett AR Duncan BB Couper DJ Tielsch JM Klein BEK Hubbard LD Retinal arteriolar narrowing and risk of coronary heart disease in men and women: The Atherosclerosis Risk in Communities Study Journal of the American Medical Association 2002 287 9 1153 1159 10.1001/jama.287.9.1153 11879113

[B24] Ikram MK De Jong FJ Van Dijk EJ Prins ND Hofman A Breteler MMB De Jong PTVM Retinal vessel diameters and cerebral small vessel disease: The Rotterdam Scan Study Brain 2006 129 1 182 188 16317022 10.1093/brain/awh688

[B25] Kwa VIH Van der Sande JJ Stam J Tijmes N Vrooland JL Retinal arterial changes correlate with cerebral small-vessel disease Neurology 2002 59 10 1536 1540 12451193 10.1212/01.wnl.0000033093.16450.5c

[B26] May GS Eberlein KA Furberg CD Passamani ER DeMets DL Secondary prevention after myocardial infarction: A review of long-term trials Progress in Cardiovascular Diseases 1982 24 4 331 352 10.1016/0033-0620(82)90010-X 6119737

[B27] Troche CJ Tacke J Hinzpeter B Danner R Lauterbach KW Cost-effectiveness of primary and secondary prevention in cardiovascular diseases European Heart Journal 1998 19 SUPPL C C59 C65 9597427

[B28] Katz S Akpom CA A measure of primary sociobiological functions International Journal of Health Services 1976 6 3 493 508 10.2190/UURL-2RYU-WRYD-EY3K 133997

[B29] Teng EL Chui HC The Modified Mini-Mental State (MMS) examination Journal of Clinical Psychiatry 1987 48 8 314 318 3611032

[B30] Ruff RM Light RH Parker SB Levin HS Benton controlled Oral Word Association Test: Reliability and updated norms Archives of Clinical Neuropsychology 1996 11 4 329 338 14588937

[B31] Brandt J Benedict R Hopkins verbal learning test - revised 2001 Inc PAR. Lutz, Fla 10.1076/clin.13.3.348.1749 10726605

[B32] Strauss E Sherman M Spreen O A compendium of Neuropsychological Tests 2006 3 New York: Oxford University Press

[B33] Radloff LS Teri L Use of the Center for Epidemiological Studies-Depression Scale with older adults Clinical Gerontologist 1986 5 1-2 119 136

[B34] Pohjasvaara T Leskelä M Vataja R Kalska H Ylikoski R Hietanen M Leppävuori A Kaste M Erkinjuntti T Post-stroke depression, executive dysfunction and functional outcome European Journal of Neurology 2002 9 3 269 275 10.1046/j.1468-1331.2002.00396.x 11985635

[B35] Stroop JR Studies of interference in serial verbal reactions Journal of Experimental Psychology 1935 18 6 643 662

[B36] D'Elia L Color Trails Test: Professional Manual 1996 Odessa, FL: Psychological Assessment Resources

[B37] Erkinjuntti T Inzitari D Pantoni L Wallin A Scheltens P Rockwood K Roman GC Chui H Desmond DW Research criteria for subcortical vascular dementia in clinical trials Journal of Neural Transmission, Supplement 2000 59 23 30 10.1007/978-3-7091-6781-6_4 10961414

[B38] Hachinski V Iadecola C Petersen RC Breteler MM Nyenhuis DL Black SE Powers WJ DeCarli C Merino JG Kalaria RN National Institute of Neurological Disorders and Stroke-Canadian Stroke Network vascular cognitive impairment harmonization standards Stroke 2006 37 9 2220 2241 10.1161/01.STR.0000237236.88823.47 16917086

[B39] Evans AC The NIH MRI study of normal brain development NeuroImage 2006 30 1 184 202 10.1016/j.neuroimage.2005.09.068 16376577

[B40] Hubbard LD Brothers RJ King WN Clegg LX Klein R Cooper LS Sharrett AR Davis MD Cai J Methods for evaluation of retinal microvascular abnormalities associated with hypertension/sclerosis in the Atherosclerosis Risk in Communities Study Ophthalmology 1999 106 12 2269 2280 10.1016/S0161-6420(99)90525-0 10599656

[B41] Knudtson MD Lee KE Hubbard LD Wong TY Klein R Klein BEK Revised formulas for summarizing retinal vessel diameters Current Eye Research 2003 27 3 143 149 10.1076/ceyr.27.3.143.16049 14562179

[B42] Verluis CE van der Mast RC van Buchem MA Bollen ELEM Blauw GJ Eekhof JAH van der Wee NJA de Craen AJM Shepherd J Cobbe SM Progression of cerebral white matter lesions is not associated with development of depressive symptoms in elderly subjects at risk of cardiovascular disease. The PROSPER Study International Journal of Geriatric Psychiatry 2006 21 4 375 381 10.1002/gps.1477 16534770

[B43] Van Den Heuvel DMJ Admiraal-Behloul F Ten Dam VH Olofsen H Bollen ELEM Murray HM Blauw GJ Westendorp RGJ De Craen AJM Van Buchem MA Different progression rates for deep white matter hyperintensities in elderly men and women Neurology 2004 63 9 1699 1701 15534259 10.1212/01.wnl.0000143058.40388.44

[B44] Patton N Aslam T MacGillivray T Pattie A Deary IJ Dhillon B Retinal vascular image analysis as a potential screening tool for cerebrovascular disease: A rationale based on homology between cerebral and retinal microvasculatures Journal of Anatomy 2005 206 4 319 348 10.1111/j.1469-7580.2005.00395.x 15817102 PMC1571489

[B45] Bush TL Riedel D Screening for total cholesterol: Do the National Cholesterol Education Program's recommendations detect individuals at high risk of coronary heart disease? Circulation 1991 83 1287 1293 2013146 10.1161/01.cir.83.4.1287

